# The rhizosphere revisited: root microbiomics

**DOI:** 10.3389/fpls.2013.00165

**Published:** 2013-05-30

**Authors:** Peter A. H. M. Bakker, Roeland L. Berendsen, Rogier F. Doornbos, Paul C. A. Wintermans, Corné M. J. Pieterse

**Affiliations:** Plant-Microbe Interactions, Department of Biology, Faculty of Science, Utrecht UniversityUtrecht, Netherlands

**Keywords:** plant roots, microbial communities, extended phenotype, *Arabidopsis thaliana*, *Pseudomonas* spp

## Abstract

The rhizosphere was defined over 100 years ago as the zone around the root where microorganisms and processes important for plant growth and health are located. Recent studies show that the diversity of microorganisms associated with the root system is enormous. This rhizosphere microbiome extends the functional repertoire of the plant beyond imagination. The rhizosphere microbiome of *Arabidopsis thaliana* is currently being studied for the obvious reason that it allows the use of the extensive toolbox that comes with this model plant. Deciphering plant traits that drive selection and activities of the microbiome is now a major challenge in which *Arabidopsis* will undoubtedly be a major research object. Here we review recent microbiome studies and discuss future research directions and applicability of the generated knowledge.

## INTRODUCTION

Ever since Lorenz Hiltner, more than a century ago, defined the rhizosphere as the soil compartment influenced by plant roots (Hiltner, 1904; Hartmann et al., 2008), this hotspot for microbial interactions and activities has received ample attention from scientists in different disciplines. Also the above ground plant surface, the so-called phyllosphere, harbors microbial communities that have more recently been studied in detail (Vorholt, 2012). The microbial activity in the rhizosphere is essential for plant functioning as it assists the plant in nutrient uptake and offers protection against pathogen attack (Berendsen et al., 2012). Microbiological studies in the soil environment are hampered by the fact that the largest proportion of soil bacteria as yet cannot be cultured (Amann et al., 1995; Kent and Triplett, 2002; Doornbos et al., 2012). However, developments in metagenomics provide a more complete picture of the rhizosphere microbiome (Leveau, 2007; Sorensen et al., 2009; Hirsch and Mauchline, 2012). Thus the microbial players in the rhizosphere are on their way to be exposed and, perhaps more importantly, transcriptomic studies of the microbiome have been initiated to reveal microbial activities in complex environments (Urich et al., 2008; Gosalbes et al., 2012; Jansson et al., 2012). Unraveling processes that drive selection and activities of the rhizosphere microbiome will open up new avenues to manipulate crop health and yield. In this paper, we review and discuss recent developments in rhizosphere microbiome studies.

## THE RHIZOSPHERE EFFECT

**FIGURE 1:**
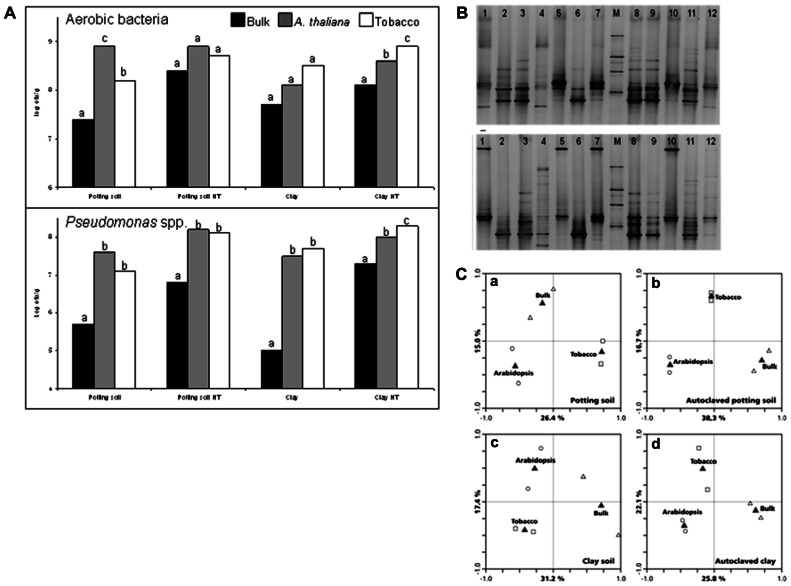
**(A)** Numbers (log cfu g^-^^1^) of culturable aerobic bacteria and *Pseudomonas* spp. in bulk (black bars) and rhizosphere soil of *Arabidopsis* (gray bars) and Tobacco (white bars). Plants were grown for 7 weeks on a potting soil–sand mixture or a clay soil, which were either untreated or autoclaved twice heat treatment (HT) before planting. Different letters indicate significant differences within each soil type. **(B)** Denaturing gradient gel electrophoresis (DGGE) profile showing the *Pseudomonas *spp. community structure from bulk soils (*top gel*: potting soil; *bottom gel*: clay soil), and the rhizospheres of *Arabidopsis* and tobacco grown on these soils. M, reference marker; lanes 1 and 10, *Arabidopsis* rhizosphere grown on non-autoclaved soil; lanes 2 and 6, autoclaved bulk soil; lanes 3 and 9, tobacco rhizosphere grown on autoclaved soil; lanes 4 and 12, non-autoclaved bulk soil; lanes 5 and 7, tobacco rhizosphere grown on non-autoclaved soil; lanes 8 and 11, *Arabidopsis* rhizosphere grown on non-autoclaved soil. **(C)** Ordination biplot generated by redundancy analysis (RDA) of *Pseudomonas*-specific DGGE fingerprints of bulk soil and the rhizospheres of *Arabidopsis* and tobacco grown on **(a)** potting soil–sand mixture; **(b)** autoclaved potting soil–sand mixture; **(c)** clay soil; **(d)** autoclaved clay soil. Open triangles, bulk; open circles, *Arabidopsis* rhizosphere; open squares, tobacco rhizosphere; gray triangles, centroid position of variables.

Compared to non-rooted bulk soil, the soil compartment directly around the plant root contains much larger populations of microorganisms (Foster et al., 1983). The increased microbial numbers and activities in the rhizosphere are due to the release of large amounts of organic carbon by the plant roots (Walker et al., 2003; Bais et al., 2006; Hartmann et al., 2009). In their extensive review, Jones et al. (2009) describe loss of root cap and border cells, insoluble mucilage, soluble root exudates, volatile organic carbon, flow of carbon to root associated symbionts, and death and lysis of root cells as the major processes of rhizodeposition. Soil microorganisms are chemotactically attracted to the plant root exudates, after which they proliferate in this carbon rich environment (Lugtenberg and Kamilova, 2009). Carbon limitation could be demonstrated in bulk soil but not in the rhizosphere using *Pseudomonas fluorescens* strains carrying carbon-limitation reporter systems (Van Overbeek et al., 1997; Koch et al., 2001). Given the fact that plant root exudates differ between plant species (Rovira, 1969), differences in rhizosphere microbiomes of different plant species are to be expected. Indeed plant-specific microbial communities could be isolated from roots in studies comparing, for example, wheat, ryegrass, bentgrass, and clover (Grayston et al., 1998), or wheat and canola (Germida et al., 1998). Also within a specific bacterial group like fluorescent *Pseudomonas* spp., plant species-specific rhizosphere populations could be isolated (Glandorf et al., 1993; Lemanceau et al., 1995). More recent studies, in which the rhizosphere microbiomes were characterized based on direct extraction of total community DNA, also provide strong evidence for plant species-specific microbiomes (Miethling et al., 2000; Smalla et al., 2001; Kirk et al., 2005; Inceoglu et al., 2013). The roots of wheat, maize, rape, and barrel clover were shown to carry different bacterial communities as a consequence of assimilation of root exudates (Haichar et al., 2008). Bacterial community structures in field grown potato rhizospheres were affected by the growth stage of the plant (Inceoglu et al., 2013). Also at the genotype level within a plant species, specificity of the rhizosphere microbiome has been described (Micallef et al., 2009a, b; Weinert et al., 2011). Micallef et al. (2009b) used *A. thaliana* and showed that the rhizosphere of this model plant mediates a significant change in the bacterial community relative to the bulk soil. To illustrate the rhizosphere effect we compared rhizosphere bacterial communities of tobacco and *A. thaliana* grown on a potting and a clay soil. In **Figure 1A** total bacterial counts on 1/10 strength tryptic soy agar (TSA) and counts of fluorescent pseudomonads on Kings medium B agar (KB) in bulk soil and in the rhizospheres of *A. thaliana* Col-0 and tobacco are presented. The rhizosphere effect is exemplified by the observation that numbers in the rhizosphere are about 10- to 100-fold higher compared to the numbers in bulk soil for both plant species. In **Figures 1B, C** , *Pseudomonas*-specific denaturing gradient gel electrophoresis (DGGE) profiles are shown and compared in a redundancy analysis. For both tobacco and *A. thaliana*, rhizosphere *Pseudomonas* communities are different from those in the bulk soil, and the communities differ between the plant species. In two recent papers the *A. thaliana* root microbiome has been described in detail using pyrosequencing of 16S rRNA gene amplicons (Bulgarelli et al., 2012; Lundberg et al., 2012). Whereas differences between bacterial communities in bulk soil and the rhizosphere were observed in these studies, their focus was on the endophytic compartment. Inside the root, the microbiome clearly differed from the bulk soil and was enriched in Actinobacteria and Proteobacteria (Bulgarelli et al., 2012; Lundberg et al., 2012).

## RECRUITMENT OF THE RHIZOSPHERE MICROBIOME

The rhizosphere bacterial community is recruited from the main reservoir of microorganisms present in soil (Normander and Prosser, 2000; De Ridder-Duine et al., 2005; Berg and Smalla, 2009). Thus the soil is an important factor in shaping the rhizosphere microbiome (Garbeva et al., 2008; Lundberg et al., 2012). As described in the previous section, plant genotype is also a driving force for the selection of specific elements from the bulk soil microbial community. Furthermore, when under attack, plants seem to actively select specific elements of their bacterial rhizosphere microflora. This is most clearly observed in so-called disease suppressive soils, in which disease will not develop despite the presence of a virulent pathogen and a susceptible plant. Disease suppressiveness is due to microbial activity and usually needs an outbreak of disease to develop (Mazzola, 2002). A well-studied example is take-all decline (TAD), which develops in continuous wheat cultures after a severe outbreak of the take-all disease caused by *Gaeumannomyces graminis* var. *tritici* (Weller et al., 2002). Heat treatment abolishes suppressiveness and the suppressiveness of TAD soil is transferable to a disease conducive soil by mixing small quantities of decline soil through conducive soil. Under continuous wheat cropping, a specific group of fluorescent pseudomonads that produce 2,4-diacetylphloroglucinol (DAPG) is enriched in the rhizosphere and these bacteria appear to be responsible for TAD (Raaijmakers and Weller, 1998). Additional bacterial taxa that may be involved in TAD have more recently been identified using 16S rRNA-based techniques (Sanguin et al., 2009; Schreiner et al., 2010). The specific selection of plant protecting bacteria in the rhizosphere under pathogen attack is supported by a recent study of Mavrodi et al. (2012). They observed that under irrigation the wheat rhizosphere recruits DAPG producing pseudomonads, whereas under dry conditions phenazine producing pseudomonads are recruited. Under irrigated conditions *G. graminis *var. *tritici* is the major soil borne pathogen of wheat, whereas under dry conditions *Rhizoctonia solani* is the main problem. Strikingly *G. graminis* var. *tritici* is more sensitive to DAPG, whereas *R. solani* is more sensitive to phenazines. Thus, under conditions that favor a specific pathogen, antagonists that are most effective against this pathogen are selected by the plant. Also for other disease suppressive soils specific elements of the microbiome have been identified that are associated with suppressiveness. In a *Fusarium* wilt suppressive soil the production of redox-active phenazines by fluorescent pseudomonads and competition for carbon by non-pathogenic *Fusarium oxysporum* have a synergistic effect that may establish suppressiveness (Mazurier et al., 2009). In a soil suppressive to potato common scab a microbial consortium that is associated with suppressiveness was identified (Rosenzweig et al., 2012). For a soil suppressive to black root rot of tobacco, caused by *Thielaviopsis basicola*, several bacterial taxa, including *Pseudomonas*, *Azospirillum*, *Gluconacetobacter*, *Burkholderia*, *Comamonas*, and Sphingomonadaceae, were shown to be more prevalent in the suppressive than in the conducive soil (Kyselkova et al., 2009). To identify bacteria involved in soil suppressiveness against *R. solani*, Mendes et al. (2011) used PhyloChip analysis, which allows simultaneous detection of ~60,000 bacterial and archaeal operational taxonomic units (OTUs). Over 33,000 OTUs were detected in the rhizospheres of sugar beet grown in *R. solani* suppressive and conducive soil. Taxa that were more abundant in suppressive soil and in a mixture of conducive soil with 10% suppressive soil than in conducive soil, but also more abundant in suppressive soil amended with *R. solani* than in suppressive soil without the pathogen, were identified. Seventeen taxa belonging to the β-proteobacteria, γ-proteobacteria, and the firmicutes were closely associated with disease suppressiveness (Mendes et al., 2011). In all disease suppressive soils mentioned here, consortia of antagonistic microorganisms seem to be recruited by the rhizosphere under pathogen attack.

Not only attack by soil borne pathogens results in the recruitment of beneficial microbes in the rhizosphere. Foliar feeding of aphids on pepper plants reduced disease development caused by the bacterial pathogen *Xanthomonas axonopodis *pv*. vesicatoria* (Lee et al., 2012). Aphid infestation resulted in increased population densities of the plant beneficial *Bacillus subtilis*, whereas it reduced rhizosphere populations of plant pathogenic *Ralstonia solanacearum*. Similar results were found for whitefly infestation of pepper plants, leading to increased resistance to pathogens and to changes in the rhizosphere microbiome (Yang et al., 2011). In the aphid and whitefly systems it would be interesting to investigate possible recruitment of rhizobacteria that produce insecticidal toxins, a feature of certain rhizosphere pseudomonads that was recently reported (Pechy-Tarr et al., 2013; Ruffner et al., 2013) *A. thaliana* plants exposed to methyl jasmonate showed a shift in their bacterial rhizosphere microbiome, including taxa that are associated with disease suppression, based on 16S rRNA gene amplicon pyrosequencing (Carvalhais et al., 2013). However, in a study by Doornbos et al. (2011), leaf application of jasmonic acid did not significantly affect the rhizosphere bacterial community of *A. thaliana*, based on DGGE analysis of 16S rRNA gene amplicons. Rudrappa et al. (2008) showed that *A. thaliana* plants infected by the bacterial leaf pathogen *P. syringae* pv. *tomato*, secrete elevated levels of malic acid in the rhizosphere. Malic acid stimulates binding to roots and biofilm formation on roots by *Bacillus subtilis* strain FB17, a beneficial microbe that can induce systemic resistance against diseases. Thus the plant benefits from protection against disease by the bacteria and in turn provides the bacteria with a more favorable environment. The recruitment of FB17 was recently shown to be mediated by root responses triggered by pathogen-derived microbe-associated molecular patterns in the leaves. Early suppression of defense genes by FB17 was postulated to facilitate colonization of this *Bacillus subtilis* strain on *A. thaliana* roots (Lakshmanan et al., 2012). Induced systemic resistance by *P. putida* KT2440 in *A. thaliana* is related to as yet unknown compounds in the root exudate that are modulated by the bacterium (Matilla et al., 2010). Thus not only pathogenic and symbiotic microorganisms seem to modulate host immunity to their own benefit, but also plant beneficial microorganisms seem to use this strategy (Zamioudis and Pieterse, 2012).

Drought stress is also a shaping factor for the rhizosphere microbiome. Drought-sensitive pepper plants that were grown under desert farming selected for a root microbiome that was enriched for bacteria that can increase photosynthesis and plant biomass production under drought stress (Marasco et al., 2012). Soil nitrogen availability influenced rhizosphere microbial communities of *Medicago truncatula* only in the presence of the plant, and it was suggested that the adaptive strategy of the plant to environmental constraints is a major factor in shaping the rhizosphere microbiome (Zancarini et al., 2012).

Root exudates play an important role in shaping the rhizosphere microbiome. In the rhizosphere of maize, exudation of the benzoxazinone DIMBOA (2,4-dihydroxy-7-methoxy-1,4-benzoxazin-3-one) resulted in increased population densities of a *P. putida* strain with plant beneficial characteristics (Neal et al., 2012). In *A. thaliana*, active exudation of phytochemicals mediated by ABC (ATP-binding cassette) transporters was demonstrated (Badri et al., 2012). In the absence of the plant, blends of collected *A. thaliana* root exudates modulated the soil microbiome. Phenolic compounds in the root exudates were suggested to act as specific substrates and signals for soil bacteria (Badri et al., 2013). Plant age affects rhizosphere bacterial communities of *A. thaliana*, suggested to be due to changes in root exudation (Micallef et al., 2009a). In an elegant study by Chaparro et al. (2013), combining metatranscriptomics and metabolomics, a strong correlation was observed between compounds released from the roots at different stages of plant development and the expression of microbial genes involved in metabolism of specific compounds.

Overall, evidence is accumulating that plants shape their rhizosphere microbiome to their own benefit, making sophisticated use of the functional repertoire of the microbiome.

## ACTIVATION OF MICROBIOME FUNCTIONS

Next to recruitment of specific soil microbes into the rhizosphere microbiome, plant roots also influence specific functions of the microbiome. Quorum sensing, regulation of microbial gene expression in response to cell density, is an important mechanism to regulate microbial activities. Such activities include antibiotic production, biofilm formation, conjugation, motility, symbiosis, and virulence (Miller and Bassler, 2001). This regulatory mechanism is not only important within a bacterial population but also between bacterial populations (Pierson and Pierson, 2007; Hosni et al., 2011). Interkingdom communication based on quorum sensing signaling molecules, *N*-acyl homoserine lactone (AHL) signals, has also been reported. Proteome analysis revealed that *M. truncatula* responds significantly to AHLs from both symbiotic and pathogenic bacteria (Mathesius et al., 2003). AHL signal molecules produced by *Serratia liquefaciens *and *P. putida* in the rhizosphere of tomato, protected the tomato plants against the fungal leaf pathogen *Alternaria alternata*, through the induction of systemic resistance (Schuhegger et al., 2006). Similarly, growth and disease resistance of *A. thaliana* are modulated by AHLs (Von Rad et al., 2008; Schikora et al., 2011; Liu et al., 2012; Schenk et al., 2012). Interkingdom communication can also involve effects of eukaryotes on bacterial gene expression. Plants can effectively interfere with quorum sensing in bacteria by producing so called AHL mimics (Teplitski et al., 2000; Gao et al., 2003). Thus there seems to be a plant-mediated fine tuning of bacterial gene expression in the rhizosphere. Microarray-based transcriptomic profiling of specific bacteria in response to root exudates of axenically grown plants has been used to identify genes in* Pseudomonas* (Mark et al., 2005) and *Bacillus amyloliquefaciens* (Fan et al., 2012) that are involved in plant microbe interactions. Using a similar approach, effects of phosphate availability on transcriptional responses of *Pseudomonas* in the rhizosphere of *Lolium perenne* was investigated (Zysko et al., 2012). All these studies show that there is a significant impact of root exudates on bacterial gene expression. The studies by Okubara and Bonsall (2008) and Kwak et al. (2012) focused on effects of host cultivar on the production of DAPG by fluorescent pseudomonads. The production of this antifungal metabolite, that plays a central role in TAD, depends on the genotypes of both the plant and the bacterial strain involved in the interaction. Effects of pathogen infection on gene expression and functional diversity has been the focus of several studies. Infection of wheat roots by *G. graminis* var. *tritici* changed gene expression of *P. fluorescens* Pf29Arp (Barret et al., 2009). Strain Pf29Arp was suggested to show an adaptive response to the so called pathorhizosphere of necrotic roots. In the rhizosphere of strawberry, infection with *Verticillium dahliae* increased hydrogen cyanide (HCN) biosynthesis gene expression in *Pseudomonas* sp. LBUM300 (DeCoste et al., 2010). HCN production by beneficial rhizobacteria has been suggested as a mechanism of biological control, and thus this study suggests that upon root pathogen attack such biocontrol activity is stimulated. Even stronger evidence that suggests up-regulation of antifungal activity upon pathogen attack comes from an elegant study by Jousset et al. (2011). They demonstrated in a split root system that infection of barley roots with *Pythium ultimum* on one side of the system, enhanced *phlA* gene expression, required for DAPG production, in *P. fluorescens* CHA0 that colonized the other side of the root system. Root exudation of fumaric acid, *p*-coumaric acid and vanillic acid was increased in *Pythium* infected plants and these phenolic acids increase *phlA* gene expression in a dose-dependent manner (Jousset et al., 2011). Thus plants seem to respond to pathogen infection by systemic signaling leading to enhanced biocontrol activity in the microbiome.

## PERSPECTIVE

Exciting new insights in interkingdom signaling in the rhizosphere and the resulting effects on plant performance have emerged during the last decade. *A. thaliana* has been the model system of choice in several recent studies (Doornbos et al., 2011; Schwachtje et al., 2011; Bulgarelli et al., 2012; Lundberg et al., 2012; Van de Mortel et al., 2012) because a large number of accessions and well characterized mutants are readily available, and transcriptomic and metabolomic analyses are standard procedure for this plant species. Revealing the composition of the microbiome and unraveling the metatranscriptome will certainly help to shed light on the very dark rhizosphere environment. However, the rhizosphere is a dynamic environment in which the microbiome will rapidly evolve in space and time. Obviously, to date many rhizosphere metagenomic studies have focused on a single or a few time points and most studies do not take spatial dynamics into account. Metabolic profiling of living microbial colonies facilitates studying spatiotemporal dynamics of metabolite production in microbial communities (Moree et al., 2012; Watrous et al., 2012). The nanospray desorption electrospray ionization (nano-DESI) mass spectrometry technology used in these studies allows for direct sampling from plant surfaces (Traxler and Kolter, 2012) and is thus a promising development for rhizosphere studies. Given the rapid technological developments, the editors of the classic book “Plant roots: the hidden half” (Eshel and Beeckman, 2013), may want to look for a new title for the next edition.

## Conflict of Interest Statement

The authors declare that the research was conducted in the absence of any commercial or financial relationships that could be construed as a potential conflict of interest.
